# Artificial Intelligence Improves Apical Four-Chamber Window Quality in Experienced but Not Novice Users

**DOI:** 10.7759/cureus.103851

**Published:** 2026-02-18

**Authors:** Jacob Lenning, Corey L Garrison, Aaron R Mahoney, Paul F Thanel

**Affiliations:** 1 Emergency Medicine, Western Michigan University Homer Stryker M.D. School of Medicine, Kalamazoo, USA

**Keywords:** artificial intelligence, medical education research, pocus (point of care ultrasound), point-of-care echocardiography, ultrasound education

## Abstract

Introduction: The most current point-of-care ultrasound (POCUS) machines incorporate artificial intelligence (AI) features to assist users in obtaining cardiac windows. Prior studies have focused on evaluating the helpfulness of AI acquisition assistance for novice users. This study sought to determine the immediate effect of AI assistance on acquisition time and image quality of apical four-chamber (A4C) cardiac ultrasound windows obtained by both novice and experienced users.

Methods: Fourteen novices with limited POCUS training during medical school and 10 experienced second- and third-year emergency medicine residents recorded A4C windows with and without AI assistance on three standardized patients in randomized order. Acquisition time in seconds was compared with the Mann-Whitney U test. Chi-square analysis was used to compare the proportion of recordings demonstrating each of the three image quality criteria: all essential structures visible, correct image plane, and proper probe placement.

Results: The median (interquartile range) acquisition time was longer with AI assistance in both user groups, though the difference was only significant for the novice users (136 (109) seconds; 75 (66); p < .01), not for the experienced users (98 (132); 66 (47); p = .18). All A4C quality criteria were significantly more likely by experienced than novice users. For experienced users, the visibility of all essential structures trended more likely with AI assistance (0.87 (0.70, 0.95); 0.67 (0.49, 0.81); p = .06) and the correct imaging plane was significantly more likely with AI assistance (0.60 (0.42, 0.75); 0.37 (0.22, 0.55); p = .03). There was no difference in proper probe placement with AI assistance for experienced users. No significant differences in image quality criteria were observed in the novice user subgroup.

Conclusion: The results provide strong evidence that the immediate effect of AI assistance was associated with longer acquisition times in novice users obtaining A4C windows, with a similar trend among experienced users. AI assistance was also associated with a higher proportion of quality criteria amongst the experienced users, but not the novice users. Therefore, medical educators should consider the experience level of POCUS learners when incorporating AI acquisition features into education and clinical practice.

## Introduction

The most current ultrasound (US) machines incorporate artificial intelligence (AI) features to assist and guide users in obtaining cardiac windows by displaying real-time animations of suggested hand movements to improve image quality. The evidence supporting the use of these features in medical education continues to grow.

In one study, inexperienced internal medicine residents who were randomly assigned to practice cardiac POCUS with AI-capable devices for two weeks demonstrated greater image quality, less time to acquire images, and improved ability to identify reduced ejection fraction on a final assessment compared to controls [1]. In another study, inexperienced medical students randomly assigned to practice with AI-capable devices as an alternative to standard instruction demonstrated improved image quality in the apical four- and five-chamber cardiac views with slower acquisition times compared to controls [2]. An additional study of inexperienced medical students demonstrated noninferiority in apical four-chamber (A4C) cardiac window quality and no significant difference in acquisition time between the AI and the traditional instruction group [3]. Other prior studies have also demonstrated that the use of AI deep-learning algorithms can assist inexperienced users, such as resident physicians and nursing staff, in acquiring echocardiograms with diagnostic quality [4-7].

The existing literature suggests that AI-capable POCUS devices may assist novices in obtaining high-quality images while supporting skill development. However, no prior study has evaluated the immediate effect of AI assistance in both novice and experienced users. The immediate effect is important to characterize amongst learners of varying experience levels to identify which users may benefit most from AI assistance when performing POCUS studies in clinical environments. Therefore, the objective of this study was to evaluate the immediate effect of AI assistance on acquisition time and image quality of A4C cardiac ultrasound windows obtained by both novice and experienced users. Understanding the relationship between user experience and the immediate effect of AI assistance will serve to guide the use of these features for A4C window acquisition in clinical settings when POCUS examinations are performed in isolation with time limitations.

## Materials and methods

Study participants

Fourteen novices consisting of 12 first-year emergency medicine residents with two fourth-year medical students and 10 experienced second- and third-year emergency medicine (EM) residents from a single residency volunteered for the study. The institutional review board (IRB) at Western Michigan University Homer Stryker M.D. School of Medicine (WMed) in Kalamazoo, MI determined that the study met the criteria for an exemption. 

Equipment

The EchoNous Kosmos Torso-One Probe (Redmond, WA) was used. AI assistance features included auto-labeling of cardiac structures, auto-grading of image quality, and real-time guidance recommendations for image quality improvement.

Study protocol

The novice users received a 10-minute lecture and a 10-minute hands-on teaching session. The experienced participants had completed two to three years of a standard EM residency POCUS curriculum [7,8]. Participants acquired A4C windows on three different standardized patients, both with and without AI assistance. The standardized patients included two female patients and one male patient between 45 and 55 years of age without known cardiac disease. The order of AI usage was randomly determined by IRB staff in a permuted block fashion, and participants never examined the same patient twice in succession. The investigator adjusted the gain and depth; otherwise, no assistance was provided. Participants notified the investigator when the best possible view had been achieved, and the investigator saved a six-second video clip.

Outcome measures

Acquisition time was defined as the total number of seconds the probe was contacting the standardized patient. Participants were unaware that the acquisition time was measured. Image quality was assessed by observing for three specific criteria: (i) essential structures visible (both atria and ventricles with mitral and tricuspid values), (ii) correct imaging plane (left ventricle bullet-shaped without rounded, foreshortened appearance and without visualization of the aortic outflow tract), and (iii) proper probe placement (indicator towards the left side of the heart with the septum centered and straight up and down).

Power analysis

An estimated sample size of 60 was needed to detect a 25-second difference in median acquisition time with a standard deviation of 30 at a 0.05 level of significance (alpha) and with 0.80 power (1-beta).

Data analysis

Acquisition times demonstrated rightward skew; therefore, median values and interquartile ranges were determined, and Mann-Whitney U tests were performed. The investigators reviewing image quality were blinded to AI use. One POCUS fellowship-trained physician and two senior resident physicians independently rated image quality. Each reviewer tabulated their observations as “yes” or “no”. Any disagreements were reviewed by the committee for accuracy. The final value for each observation used for statistical analysis was that which two of the three investigators initially indicated. No changes were made from this determination upon the committee review. Relative proportions with 95% confidence intervals (CI; Wilson score) for the three image quality criteria with and without AI were determined. Pearson’s chi-square test and McNemar’s test for repeated-measures were used for comparison testing. All statistical tests were conducted using R Studio (Boston, MA). The level of significance (alpha) for all statistical tests was .05.

## Results

A total of 143 A4C windows were recorded. One recording was missing and excluded from paired analysis. The median (interquartile range) acquisition time for novice users (104 (89) seconds) was longer than for experienced users (74 (74)), inclusive of all recordings (p = 0.04; Table 1). The median acquisition time was longer with AI assistance in both user groups, though the difference was only significant for the novice users (136 (109); 75 (66); p < .01), not for the experienced users (98 (132); 66 (47); p = .18). All A4C quality criteria were significantly more likely amongst experienced than novice users (Table 2). For experienced users, the visibility of all essential structures trended more likely with AI assistance (0.87 (0.70, 0.95); 0.67 (0.49,0.81); p = .06) and the correct A4C imaging plane was significantly more likely with AI assistance (0.60 (0.42, 0.75); 0.37 (0.22, 0.55); p = .03; Table 3). There was no difference in proper probe placement with AI assistance for experienced users. No significant differences in image quality criteria were observed in the novice users (Table 3).

**Table 1:** Acquisition Time Median (interquartile range; IQR) time in seconds to acquire the apical four-chamber window with and without artificial intelligence (AI). p-values from the Mann-Whitney U test.

Study Group	n	Median (IQR) Seconds	p-Value	U-Statistic
Novice User Recordings	83	104 (89)	0.04	2000
Experienced User Recordings	60	74 (74)		
Novice Users With AI	42	136 (109)	<0.01	1400
Novice Users Without AI	41	75 (66)		
Experienced Users With AI	30	98 (132)	0.18	540
Experienced Users Without AI	30	66 (47)		

**Table 2:** Image Quality by Experience Level Proportion (95% confidence intervals) of apical four-chamber recordings meeting quality criteria by the experience level, inclusive of AI and non-AI recordings. p-values from Pearson’s chi-square test (X^2^, df = degrees of freedom).

Criteria	Proportion (95% CI)		Chi-Square Test (df=1)		
	Novice Users	Experienced Users	n	p	X^2^
Essential Structures	0.58 (0.47, 0.68)	0.77 (0.64, 0.86)	143	0.02	5.5
Imaging Plane	0.22 (0.14, 0.32)	0.48 (0.36, 0.61)		<0.01	11
Probe Placement	0.41 (0.31, 0.52)	0.73 (0.61, 0.83)		<0.01	15

**Table 3:** Image Quality With and Without Artificial Intelligence Assistance Proportion (95% confidence intervals) of apical 4-chamber recordings obtained with and without (WO) artificial intelligence (AI) meeting quality criteria. p-values from McNemar’s test for repeated measures (X^2^, df = degrees of freedom).

Group	Criteria	Proportion (95% CI)		McNemar’s Test (df =1)		
		AI	WO	n	p	X^2^
Novice Users	Essential Structures	0.57 (0.42, 0.71)	0.59 (0.43, 0.72)	41	0.80	0.067
	Imaging Plane	0.26 (0.15, 0.41)	0.17 (0.08, 0.32)		0.21	1.6
	Probe Placement	0.43 (0.29, 0.58)	0.39 (0.26, 0.54)		0.80	0.067
Experienced Users	Essential Structures	0.87 (0.70, 0.95)	0.67 (0.49, 0.81)	30	0.06	3.6
	Imaging Plane	0.60 (0.42, 0.75)	0.37 (0.22, 0.55)		0.03	4.5
	Probe Placement	0.73 (0.55, 0.86)	0.73 (0.55, 0.86)		1.00	0

## Discussion

This is the first study investigating the immediate effect of AI assistance on acquisition time and image quality in both novice and experienced users obtaining the A4C cardiac window. We observed significantly longer acquisition time among novice users utilizing AI assistance, and though the difference in acquisition time was not significant among experienced users, we suspect a larger sample size would confirm a similar trend. AI assistance was also associated with a greater proportion of the image quality criteria in experienced users, with a significant difference in the correct imaging plane and a near-significant difference in the visualization of all essential structures. However, we observed no differences in the image quality criteria amongst novice users with and without AI assistance.

Prior studies in which participants were trained in cardiac POCUS with AI assistance and then were later assessed without AI assistance reported varied effects on acquisition time [1,3]. Baum et al. reported significantly reduced A4C acquisition time among participants who trained with AI assistance compared to controls [1]. However, the AI group performed more practice attempts (median 5.5; IQR 4-10) than the control group (2; 0-4) during the two-week intervention period (p < 0.01), possibly confounding the results. In another study by Lau et al., there were no significant differences in A4C acquisition time during the initial or three-month follow-up assessments by participants who trained with AI assistance for one hour compared to those who received traditional faculty-led instruction for one hour [3]. Karni et al. performed the only prior study in which the intervention group used AI-assistance during the final assessment, somewhat similar to our study, and demonstrated longer acquisition times among the AI users obtaining multiple cardiac windows [2].

Our repeated-measures study differed significantly from these prior investigations by evaluating the immediate effect of AI assistance on A4C acquisition time in the same user as opposed to assigning users to AI and non-AI intervention groups [1-3]. Our results demonstrated that AI assistance was associated with longer acquisition time within users, not just between users. We suspect the AI assistance, particularly the auto-grading features, prompted users in our study to spend more time attempting to obtain higher quality windows. Further study is needed, but based on our results, we would hypothesize that the use of AI assistance may lead to longer acquisition times in isolated cardiac POCUS exams performed in clinical settings by any user, regardless of their experience level.

Uniquely, our study demonstrated a “leveling-up effect” or improvement in image quality among experienced users with AI assistance, which had not been previously reported in the literature. However, contrary to the previous studies, we observed no clear evidence that AI assistance was associated with improved image quality amongst novice users obtaining A4C windows [1-7]. This is despite using the same AI-capable US equipment (EchoNous Kosmos) as two of the prior studies [1,3]. Besides the repeated-measure design of our study assessing the immediate effect of AI assistance, another major difference from previous studies was the length of the training and intervention period. Most prior studies included training and intervention periods ranging from an hour to even weeks, and thereby they evaluated the effect of practicing with AI-capable US devices as opposed to the immediate effect of their use [1-7]. Only Mears et al. utilized a training period similar in length to that of our study, which was reported to be 10 minutes compared to a total of 20 minutes in our study [4]. Considering that perhaps our results were influenced by the limited training and intervention period in our study compared to others, educators should ensure novice users receive ample hands-on instruction with the AI-capable devices prior to self-study with the devices. Further investigations are needed to determine the minimum time-frame, but based on inferences from our study and those prior, we would recommend at least an hour for adequate introduction to the AI-capable devices and the targeted POCUS exams.

Furthermore, no other study performed a baseline assessment of image quality to compare the immediate effect of using AI assistance to obtain the A4C window within the same user. Three prior studies compared image quality between participants who were randomized to AI or non-AI intervention groups and all reported improved A4C image quality in the AI intervention group [1-3]. Other studies evaluated image quality by determining the proportion of A4C windows with sufficient quality for diagnostic assessment [4-7]. The proportions were wide ranging, with Mears et al. reporting only about 63% of AI-assisted A4C windows were of diagnostic quality to assess left ventricular function, but studies by Gallant et al., Narang et al., and Mor-Avi et al. all reported proportions >95% [5-7].

The importance of the baseline image quality measurements in our study is illustrated by comparing our results to the Mears et al. study of neurology learners [4]. In that study, 43% of all A4C windows obtained by users with AI assistance demonstrated recognizable structures and were scored as three or greater on the American College of Emergency Physicians (ACEP) scale, though no baseline assessment without AI was performed [3,9]. The proportion of our novice user recordings obtained with AI demonstrating all essential structures, which would also be scored three or greater on the ACEP scale, was slightly higher at 59%. However, we observed no difference in the proportion of novice user recordings with all essential structures visualized without (57%; p = .80), suggesting that AI assistance was not beneficial for the novice users in our study.

In summary, we suspect our novice users lacked the US probe manipulation motor skills needed to benefit from AI assistance. It is possible our sample of novice users differed from those of prior studies; however, our results should still encourage caution when using AI-capable US devices in medical education. POCUS instructors should ensure novice users continue to undergo personalized instruction with hands-on training, especially in clinical settings when AI-capable devices are typically used in isolated attempts. However, our results do indicate that experienced users are likely to benefit from improved A4C image quality when using AI-assisted acquisition in isolated attempts. Further study will need to validate this “leveling-up effect” in experienced users obtaining the A4C window observed in our study and should evaluate the remaining cardiac POCUS windows similarly.

Limitations

The most notable limitation in our study is the small sample size, increasing the risk of type II error. Other limitations include the relatively short training and intervention period in our study compared to that of prior studies, which could have predisposed our novices to longer acquisition time and poorer image quality. Additionally, a quantitative grading system, such as the ordinal ACEP grading scale utilized by Baum et al. or the Rapid Assessment of Competency in Echocardiography (RACE) score utilized by Lau et al., may have detected more subtle differences in image quality [1,3,9,10]. However, our grading system did detect differences in image quality between novice and experienced users, providing internal validation of the process. Finally, the repeated-measure design could have introduced learning bias as a confounding variable. Users may have remembered where to place the probe for the best quality image on their second examination of the same standardized patient. To limit learning bias, we randomized the order of AI usage.

## Conclusions

The results provide strong evidence that the immediate effect of AI assistance was associated with longer acquisition times in novice users obtaining A4C windows, with a similar trend among experienced users. AI assistance was also associated with a higher proportion of quality criteria amongst the experienced users, but not the novice users. Therefore, medical educators should consider the experience level of POCUS learners when incorporating AI acquisition features into education and clinical practice.
